# PD-L1 enhances CNS inflammation and infarct volume following experimental stroke in mice in opposition to PD-1

**DOI:** 10.1186/1742-2094-10-111

**Published:** 2013-09-09

**Authors:** Sheetal Bodhankar, Yingxin Chen, Arthur A Vandenbark, Stephanie J Murphy, Halina Offner

**Affiliations:** 1 Neuroimmunology Research, R&D-31, Portland Veterans Affairs Medical Center, 3710 SW US Veterans Hospital Road, Portland, OR 97239, USA; 2 Department of Neurology, Oregon Health & Science University, Portland, OR 97239, USA; 3 Department of Molecular Microbiology & Immunology, Oregon Health & Science University, Portland, OR 97239, USA; 4 Department of Anesthesiology & Perioperative Medicine, Oregon Health & Science University, Portland, OR 97239, USA; 5 Department of Behavioral Neuroscience, Oregon Health & Science University, Portland, OR 97239, USA

**Keywords:** Co-inhibitory pathway, Inflammatory states, MCAO, Programmed death-1 ligand 1 and 2

## Abstract

**Background:**

Stroke severity is worsened by recruitment of inflammatory immune cells into the brain. This process depends in part on T cell activation, in which the B7 family of co-stimulatory molecules plays a pivotal role. Previous studies demonstrated more severe infarcts in mice lacking programmed death-1 (PD-1), a member of the B7 family, thus implicating PD-1 as a key factor in limiting stroke severity. The purpose of this study was to determine if this protective effect of PD-1 involves either of its ligands, PD-L1 or PD-L2.

**Methods:**

Central nervous system (CNS) inflammation and infarct volume were evaluated in male PD-L1 and PD-L2 knockout (^-/-^) mice undergoing 60 minutes of middle cerebral artery occlusion (MCAO) followed by 96 hours of reperfusion and compared to wild-type (WT) C57BL/6J mice.

**Results:**

PD-L1^-/-^ and PD-L2^-/-^ mice had smaller total infarct volumes compared to WT mice. The PD-L1^-/-^ and to a lesser extent PD-L2^-/-^ mice had reduced levels of proinflammatory activated microglia and/or infiltrating monocytes and CD4^+^ T cells in the ischemic hemispheres. There was a reduction in ischemia-related splenic atrophy accompanied by lower activation status of splenic T cells and monocytes in the absence of PD-L1, suggesting a pathogenic rather than a regulatory role for both PD-1 ligands (PD-Ls). Suppressor T cells (IL-10-producing CD8^+^CD122^+^ T cells) trafficked to the brain in PD-L1^-/-^ mice and there was decreased expression of CD80 on splenic antigen-presenting cells (APCs) as compared to the WT and PD-L2^-/-^ mice.

**Conclusions:**

Our novel observations are the first to implicate PD-L1 involvement in worsening outcome of experimental stroke. The presence of suppressor T cells in the right MCAO-inflicted hemisphere in mice lacking PD-L1 implicates these cells as possible key contributors for controlling adverse effects of ischemia. Increased expression of CD80 on APCs in WT and PD-L2^-/-^ mice suggests an overriding interaction leading to T cell activation. Conversely, low CD80 expression by APCs, along with increased PD-1 and PD-L2 expression in PD-L1^-/-^ mice suggests alternative T cell signaling pathways, leading to a suppressor phenotype. These results suggest that agents (for example antibodies) that can target and neutralize PD-L1/2 may have therapeutic potential for treatment of human stroke.

## Background

Stroke is the fourth most common cause of death and a leading cause of disability in the United States alone. It is increasingly accepted that human stroke does not just create a single organ injury but induces a complex interaction between the central nervous system (CNS) and peripheral immune system. Experimental stroke in mice induces a biphasic response in the peripheral immune system characterized by an initial activation phase (6 to 22 hours) [1] followed by an immunosuppressive phase (96 hours) which is accompanied by a pronounced atrophy of the spleen and thymus [2]. Peripheral immune cells home to the brain, transmigrate over the activated endothelium and invade the damaged brain in a timed fashion [3]. The developing infarct is exacerbated by the influx of inflammatory cells and the time course and degree of accumulation of multiple inflammatory cell types in the brain has been extensively studied [4-6]. T cells may be major offenders in mediating the post-stroke inflammatory response, contributing to increased brain damage. When activated, T cells produce cytokines that initiate an inflammatory cascade involving recruitment of other inflammatory cells to sites of injury [7]. T cells are observed in the brain within hours of the ischemic insult [4,8] and T cell-deficient animals have reduced infarct size after stroke [9].

The activation of T cells via the various antigen-presenting cells (APCs) forms an integral part of the inflammatory process. This process gains importance especially since the inflammatory cells in ischemic stroke include CD4^+^ and CD8^+^ T lymphocytes, microglia, and macrophages [4]. T cell activation is a complex and multistep phenomenon and the B7 family of co-stimulatory molecules play a pivotal role in optimal activation of T cells [10]. Programmed death-1 (PD-1), a member of the B7-CD28 family, is a co-inhibitory receptor expressed by a variety of activated immune cells, including CD4^+^ and CD8^+^ T cells, natural killer T (NKT) cells, B cells, monocytes, and some dendritic cell (DC) subsets [11]. PD-L1 and PD-L2 are the two known ligands for PD-1 [12] and they have different expression patterns. While PD-L1 is found constitutively expressed on murine lymphoid cells, such as T cells, B cells, macrophages, DCs, mesenchymal stem cells, and bone marrow-derived mast cells [13], it is also expressed by non-hematopoietic cells [12,14-18]. Expression of PD-L2 on the other hand is restricted to macrophages, DCs, bone marrow-derived mast cells, and peritoneal B1 cells [12,13,19-21].

Our previous work demonstrated an increased expression of PD-1 on brain macrophages and microglial cells and PD-L1/2 on B cells from the spleen, blood, and CNS in mice after induction of experimental stroke by middle cerebral artery occlusion (MCAO). Thus, when MCAO was compared in PD-1 knockout (^-/-^) versus wild-type (WT) male mice after 96 hours of reperfusion, cortical, striatal, and total infarct volumes were significantly larger in PD-1^-/-^ versus WT with substantial recruitment of inflammatory cells from the periphery into the CNS [22], clearly implicating the role of the PD-1/PD-1 ligand (PD-L) pathway in limiting infarct volume in MCAO. Thus, the aim of the present study was to extend our previous studies and investigate the role of the PD-Ls, PD-L1 and PD-L2, in modulating severity of ischemic brain injury and associated CNS inflammation. For this purpose, PD-L1^-/-^ and PD-L2^-/-^ male mice, on a C57BL/6 background, were subjected to 60 minutes of MCAO followed by 96 hours of reperfusion and infarct volumes and immunological parameters were compared to similarly treated WT mice. Our results clearly demonstrate that PD-L1^-/-^ and PD-L2^-/-^ mice had lower total infarct volumes compared to WT mice. The immune parameters matched the stroke outcome in that the PD-L1^-/-^ and to a lesser extent PD-L2^-/-^ had reduced levels of proinflammatory activated microglia and/or infiltrating monocytes and CD4^+^ T cells in the ischemic hemispheres. There was a reduction in ischemia-related splenic atrophy accompanied by lower activation status of splenic T cells and monocytes in the absence of the PD-L1, suggesting a pathogenic rather than a regulatory role for both PD-Ls. Also, suppressor T cells (IL-10-producing CD8^+^CD122^+^ T cells) trafficked to the brain in PD-L1^-/-^ mice possibly acting as key contributors of immunoregulation. Thus, these results clearly establish a role for PD-L1 and to a lesser extent PD-L2 in increasing infarct volumes in experimental stroke, making them potential targets for future immunotherapy.

## Materials and methods

### Animals

PD-L1-deficient (PD-L1^-/-^) and PD-L2-deficient (PD-L2^-/-^) mice on a C57BL/6 background were gifts from Indira Guleria, PhD (Transplantation Research Center, Brigham and Women’s Hospital, Children’s Hospital Boston, and Harvard Medical School, Boston, MA, USA) and Arlene Sharpe, PhD (Department of Pathology, Harvard Medical School, Boston, MA, USA), respectively. Based on Dr. Guleria’s and Sharpe’s recommendations and previous publications [20,23], age-matched 8 to 12-week-old male WT C57BL/6J mice (Jackson Laboratory, Bar Harbor, ME, USA) were used as controls. Animals were randomized to treatment groups. All experiments were performed in accordance with National Institutes of Health (NIH) guidelines for the use of experimental animals, and the protocols were approved by Portland Veteran Affairs Medical Center and Oregon Health & Science University Animal Care and Use Committees.

### Middle cerebral artery occlusion (MCAO) model

All surgeries were conducted under aseptic conditions by a single surgeon who was not blinded to the animal’s genotype. Transient focal cerebral ischemia was induced in male PD-L1^-/-^, PD-L2^-/-^, and WT mice for 60 minutes by reversible right MCAO under isoflurane anesthesia followed by 96 hours of reperfusion as previously described [24]. Head and body temperature were controlled at 36.5 ± 1.0°C (mean ± SD) before, during, and after MCAO with warm water pads and a heating lamp. The common carotid artery was temporarily occluded and a 6-0 nylon monofilament surgical suture (Ethicon, Somerville, NJ, USA) with a silicone-coated (Xantopren Comfort Light, Heraeus, Hanau, Germany) tip was inserted via an external carotid artery stump distal to the internal carotid artery to the origin of the middle cerebral artery. After 60 minutes of MCAO, reperfusion was initiated by intraluminal filament withdrawal and the incision was closed with 6-0 surgical sutures (Ethicon). Each animal was then awakened and recovered in a separate cage with a warm water pad. In sham-treated mice, the filament was placed but not advanced to achieve MCAO. Occlusion and reperfusion were verified in each animal by laser Doppler flowmetry (LDF) (Model DRT4, Moor Instruments, Wilmington, DE, USA). Animals were excluded if intraischemic LDF (percentage of preischemic LDF baseline) was greater than 30%. Neurological deficit scores were determined at 1, 24, 48, 72, and 96 hours of reperfusion to confirm ischemia and the presence of ischemic injury using a 0 to 4-point scale as follows: 0, no neurological dysfunction; 1, failure to extend left forelimb fully when lifted by tail; 2, circling to the contralateral side; 3, falling to the left; and 4, no spontaneous movement or in a comatose state [24]. Any animal without a deficit at 1 hour of reperfusion was excluded from the study.

### Infarct volume analysis

Individual performing infarct volume analysis was not blinded to genotype. Mice were euthanized and brains collected at 96 hours of reperfusion for 2,3,5-triphenyltetrazolium chloride histology and then digital image analysis of infarct volume was undertaken as previously published [24]. Images were analyzed using SigmaScan Pro 5.0 (Systat Software, Inc, Point Richmond, CA, USA). To control for edema, regional infarct volume (cortex, striatum, and hemisphere) was determined by subtraction of the ipsilateral non-infarcted regional volume from the contralateral regional volume. This value was then divided by the contralateral regional volume and multiplied by 100 to yield regional infarct volume as a percentage of the contralateral region.

### Cell isolation

Splenocyte suspensions were prepared by mechanical disruption followed by using red cell lysis buffer (eBioscience, San Diego, CA, USA) according to the manufacturer’s instructions. For preparation of inflammatory cells from the brain, each mouse was perfused transcardially with 30 mL saline to exclude blood cells, the forebrain was dissected from the cerebellum and suspended in RPMI-1640 medium, and the suspension was digested with type IV collagenase (1 mg/mL, Sigma-Aldrich, St Louis, MO, USA) and DNase I (50 mg/mL, Roche, Basel, Switzerland) at 37°C for 45 minutes in a shaker at 180 times per minute. Inflammatory cells were isolated by 37% to 70% Percoll density gradient centrifugation as previously described [25]. The cells were washed twice with RPMI-1640, counted, and resuspended in stimulation medium containing 10% FCS for phenotyping.

### Analysis of cell populations by FACS

Individual performing FACS analysis was not blinded to genotype. Anti-mouse antibodies CD19 (1D3, BD Pharmingen, BD, Franklin Lakes, NJ, USA), CD45 (30-F11, Invitrogen, Carlsbad, CA, USA), CD11b (M1/70, eBioscience), MHCII (2G9, BD Pharmingen, BD), CD4 (GK1.5, BD Pharmingen, BD), CD8 (53-6.7, BD Pharmingen, BD), CD69 (H1.2F3, BD Pharmingen, BD), CD122 (TM-β1, BD Pharmingen, BD), PD-1 (RMP1-30, eBioscience), PD-L1 (MIH5, eBioscience), and PD-L2 (TY25, eBioscience) were used for the study. Single-cell suspensions were washed with staining medium (PBS containing 0.1% NaN_3_ and 2% FCS). After incubation with mAb and washing with staining buffer, propidium iodide (PI) was added to identify dead cells. The Foxp3 staining kit (eBioscience) was used according to the manufacturer’s protocol as previously described [26]. FACS data acquisition was performed on a FACSCalibur flow cytometer (BD Biosciences, San Jose, CA, USA) and data were analyzed using isotype control antibodies to set quadrants before calculating the percentage of positive cells, using FCS Express (De Novo Software, Los Angeles, CA, USA).

### Intracellular staining

Intracellular staining was visualized using a published immunofluorescence protocol [27]. Briefly, 2 × 10^6^ cells/mL were resuspended in complete medium (RPMI-1640 containing 10% FCS, 1 mM/L pyruvate, 200 μg/mL penicillin, 200 U/mL streptomycin, 4 mM/L L-glutamine, and 5 × 10^−5^ mol/L 2-β-ME), with PMA (50 ng/mL), ionomycin (500 ng/mL), and Brefeldin A (10 μg/mL, Sigma-Aldrich) for 4 hours. For intracellular IL-10 detection, a modification was followed for the immunofluorescence staining protocol [28]. Briefly, isolated leukocytes or purified cells were resuspended (2 × 10^6^ cells/mL) in complete medium and cultured with LPS (10 μg/mL) in addition to PMA (50 ng/mL), ionomycin (500 ng/mL), and Brefeldin A (10 μg/mL) (all reagents from Sigma-Aldrich) for 4 hours. Fc receptors were blocked with anti-FcRmAb (2.3G2, BD Pharmingen, BD) before cell surface staining, fixed, and permeabilized with the Fixation/Permeabilization buffer (eBioscience) according to the manufacturer’s instructions. Permeabilized cells were washed with 1X Permeabilization Buffer (eBioscience) and stained with anti-TNF-α (MP6-XT22, BD Pharmingen, BD), anti-INF-γ (XMG1.2, eBioscience), or APC-conjugated anti-IL-10 mAb (JES5-16E3, eBioscience). Isotype matched mAb served as negative controls to demonstrate specificity and to establish background TNF-α, IFN-γ, and IL-10 staining levels.

### Statistical analysis

Infarct volume data are presented as mean ± SEM. Differences in cortical, striatal, and hemispheric (total) infarct volume were determined by one-way analysis of variance (ANOVA) with post-hoc Newman-Keuls test. A Kruskal-Wallis ANOVA on ranks was conducted to evaluate differences among the three experimental groups (WT, PD-L1^-/-^, and PD-L2^-/-^) in median neurological deficit score. Post-hoc Dunn’s test was used for all pairwise comparisons against the control (WT) group following rank-based ANOVA as treatment group sizes were unequal. The multiple comparisons on ranks did not include an adjustment for ties. Statistical significance was *P* <0.05. Statistical analyses were performed using SigmaStat Statistical Software, Version 3.1 (SPSS Inc, Chicago, IL, USA). For flow data analysis and representation of three and more groups, the one-way ANOVA followed by post-hoc Tukey’s test was applied. For all tests, *P* values ≤0.05 were considered statistically significant. All values are reported as mean ± SEM. Significant differences are denoted as *P ≤0.05; **P ≤0.01; ***P ≤0.001.

## Results

### Absence of PD-1 ligands ameliorates infarct volume and reduces neurological deficits

**Figure 1:**
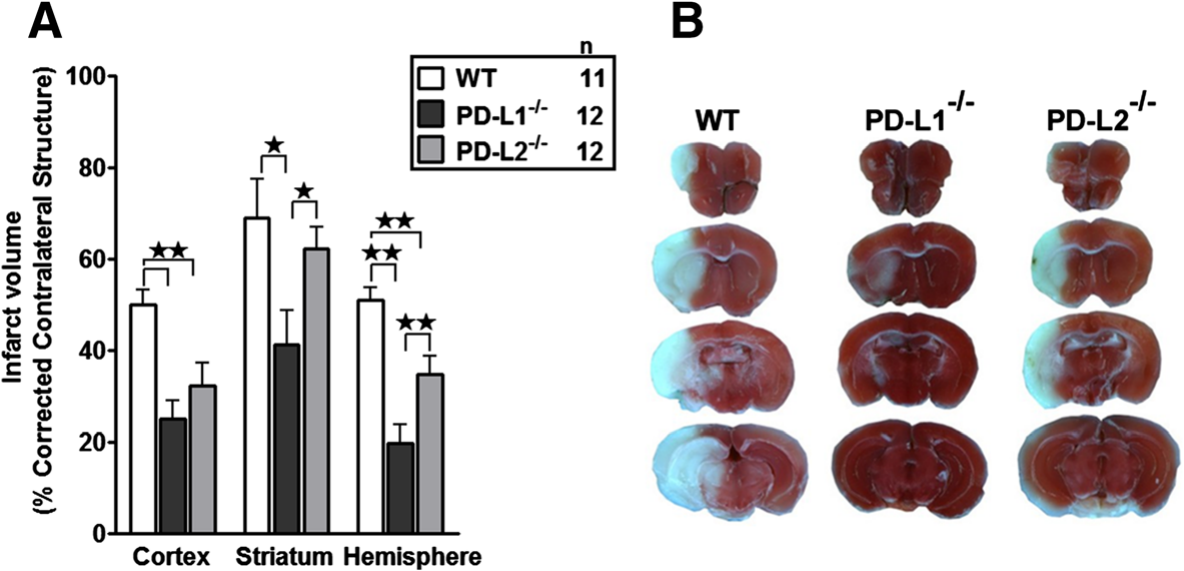
**Absence of PD-1 ligands reduces infarct volume.** Infarct volume (percentage corrected contralateral structure) in cortex, striatum, and hemisphere were determined by 2,3,5-triphenyltetrazolium chloride staining in adult male C57BL/6J wild-type (WT), PD-L1^-/-^, and PD-L2^-/-^ mice. All mice underwent 1 hour of middle cerebral artery occlusion (MCAO) followed by 96 hours of reperfusion. **(A)** PD-L1^-/-^ (n = 12) and PD-L2^-/-^ (n = 12) mice have reduced infarct volume compared to male WT mice (n = 11). Values represent mean ± SEM. *P <0.05; **P <0.01. **(B)** Representative cerebral sections showing that localization of the ischemic lesion differed among WT, PD-L1^-/-^, and PD-L2^-/-^ mice. ^-^/^-^, knockout; MCAO, middle cerebral artery occlusion; PD-1, programmed death-1; PD-L1, programmed death-1 ligand 1; PD-L2, programmed death-1 ligand 2; SEM, standard error of the mean; WT, wild-type.

Genetic deletion of either PD-L1 (25 ± 4%, *P* <0.001) or PD-L2 (32 ± 5%, *P* = 0.006) reduced cortical infarct volume when compared to male WT mice (50 ± 3%) (Figure 1A). In striatum, genetic deletion of PD-L1 (41 ± 8%, *P* = 0.024), but not PD-L2 (62 ± 5%, P = 0.502), decreased infarct volume in comparison to male WT mice (69 ± 8%) (Figure 1A). While no differences were seen in cortical infarct volume between PD-L1^-/-^ and PD-L2^-/-^ mice (*P* = 0.214), striatal infarct volume did differ between these two strains (*P* = 0.040) (Figure 1A). Compared to male WT mice (51 ± 3%), genetic deletion of either PD-L1 (20 ± 4%, *P* <0.001) or PD-L2 (35 ± 4%, *P* = 0.005) reduced hemispheric infarct volume. We also observed that hemispheric infarct volume was smaller in PD-L1^-/-^ versus PD-L2^-/-^ mice (20 ± 5% versus 35 ± 4%, *P* = 0.006). Representative cerebral sections from WT, PD-L1^-/-^, and PD-L2^-/-^ mice are shown in Figure 1B.

Distribution of neurological deficit scores within each group at each time point would suggest that loss of PD-L1 had a greater impact on decreasing, and thus improving, neurological deficit score over time than did loss of PD-L2 when compared to WT mice (Table 1). Differences in the median neurological deficit scores among the three experimental groups (WT, PD-L1^-/-^, and PD-L2^-/-^) were greater than would be expected by chance at 1 hour (*P* = 0.013), 24 hours (*P* = 0.044), 48 hours (*P* = 0.031), and 96 hours (*P* = 0.020) of reperfusion, but not at 72 hours of reperfusion (*P* = 0.062) (Table 1). A significant difference (*P* <0.05) was observed between WT and PD-L1^-/-^ groups at 1 hour and 24 hours of reperfusion (Table 1). Thus, these results demonstrate for the first time that although PD-1 limits the damage after MCAO, its ligands PD-L1 and PD-L2 enhance infarct volumes.

**Table 1:** **Neurological deficit score distribution and median scores at various reperfusion time points following 60 minutes of middle cerebral artery occlusion (MCAO) in wild-type (WT, C57BL/6J), PD-L1**^**-/-**^**, and PD-L2**^**-/- **^**mice**

**Experimental groups**	**Distribution of neurological deficit scores post-MCAO**																								
	**1 hour**					**24 hours**					**48 hours**					**72 hours**					**96 hours**				
	**0**	**1**	**2**	**3**	**4**	**0**	**1**	**2**	**3**	**4**	**0**	**1**	**2**	**3**	**4**	**0**	**1**	**2**	**3**	**4**	**0**	**1**	**2**	**3**	**4**
WT (n = 11)	0	0	4	7	0	0	4	1	6	0	0	5	3	6	0	0	5	5	1	0	0	5	2	4	0
PD-L1^-/-^ (n = 12)	0	1	10	1	0	0	9	2	1	0	0	11	0	1	0	0	11	1	0	0	0	12	0	0	0
PD-L2^-/-^ (n = 12)	0	0	6	6	0	0	3	8	1	0	0	4	8	0	0	0	7	2	3	0	0	7	1	1	3
	**Median neurological deficit scores post-MCAO**																								
	**1 hour (*P *= 0.013)**					**24 hours (*P *= 0.044)**					**48 hours (*P *= 0.031)**					**72 hours (*P *= 0.062)**					**96 hours (*P *= 0.020)**				
WT (n = 11)	3					3					2					2					2				
PD-L1^-/-^ (n = 12)	2^a^					1^a^					1					1					1				
PD-L2^-/-^ (n = 12)	2.5					2					2					1					1				

^a^*P* <0.05 compared to WT. ^-^/^-^, knockout; MCAO, middle cerebral artery occlusion; PD-L1, programmed death-1 ligand 1; PD-L2, programmed death-1 ligand 2; WT, wild-type.

### Absence of PD-L1 leads to reduced cerebral cell infiltration and inflammatory responses in the ischemic brain

**Figure 2:**
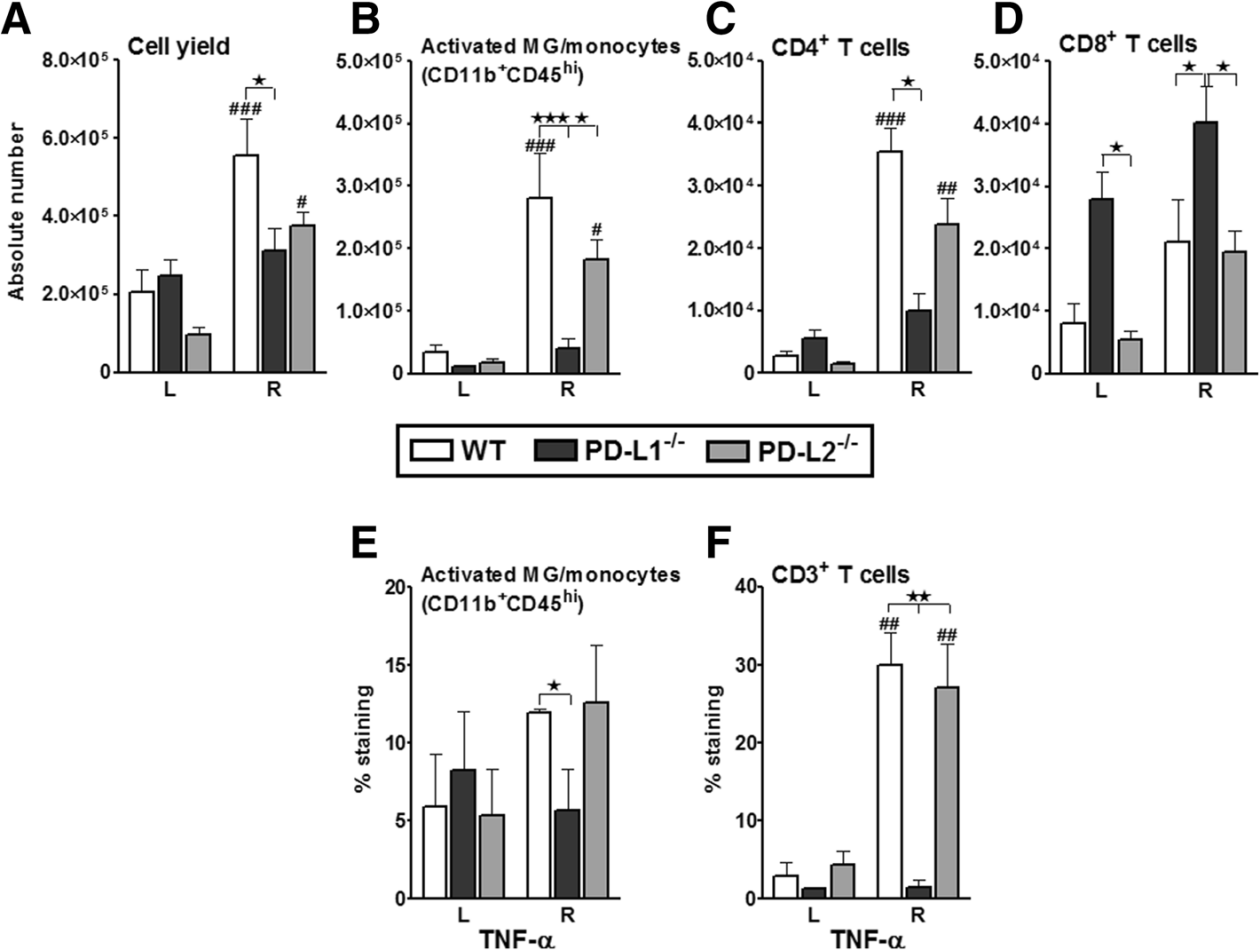
**Absence of PD-L1 reduces cerebral cell infiltration and inflammatory responses in the ischemic brain.** Ninety-six hours after MCAO, mononuclear cells were isolated from the brains of WT, PD-L1^-/-^, and PD-L2^-/-^ male mice and analyzed. **(A)** Total cell count via hemocytometer. Values represent mean numbers (± SEM) of indicated cell subsets from 9 to 11 mice per group, from three separate experiments. **(B)** CD11b^+^CD45^high^ activated microglia/monocytes. **(C)** CD4^+^ T cells and **(D)** CD8^+^ T cells were obtained from the non-ischemic (left) and ischemic (right) hemispheres of WT, PD-L1^-/-^, and PD-L2^-/-^ mice. (B,C,D) Values represent mean numbers (± SEM) of indicated cell subsets, gated on live leukocytes (by PI exclusion) from eight to nine mice per group, from at least three separate experiments. Determinations of **(E)** TNF-α production by CD11b^+^CD45^high^ activated microglia/monocytes and **(F)** TNF-α^+^CD3^+^ T cells in the non-ischemic (left) and ischemic (right) hemispheres from WT, PD-L1^-/-^, and PD-L2^-/-^ mice at 96 hours post-MCAO. Values represent mean numbers (± SEM) of indicated cell subsets from four to five mice of each group, from at least two separate experiments. Statistical analysis was performed with ANOVA followed by Tukey’s multiple comparison post-hoc test. Significant differences between sample means are indicated (#*P* ≤0.05; ##*P* ≤0.01; ###*P* ≤0.001 as compared to their respective left hemisphere and *P ≤0.05; **P ≤0.01 as compared to the ischemic right hemisphere of PD-L1^-/-^ mice post-MCAO). ^-^/^-^, knockout; ANOVA, analysis of variance; MCAO, middle cerebral artery occlusion; PD-L1, programmed death-1 ligand 1; PD-L2, programmed death-1 ligand 2; PI, propidium iodide; SEM, standard error of the mean; TNF, tumor necrosis factor; WT, wild-type.

Leukocytes are major effectors of inflammatory damage after experimental brain ischemia. To determine if the loss of the PD-Ls altered leukocyte composition in the brain after MCAO, absolute numbers of total viable leukocytes were enumerated. The ischemic (ipsilateral) hemisphere in the WT mice had a significant increase (*P* ≤0.001) in the total number of viable leukocytes as compared to the unaffected (contralateral) hemisphere (Figure 2A). While the PD-L2^-/-^ mice also demonstrated significantly higher numbers (*P* ≤0.05) of leukocytes in their ipsilateral hemispheres compared to the contralateral side, there was no difference in the numbers of viable leukocytes between the ipsilateral and contralateral hemispheres of PD-L1^-/-^ mice. The PD-L1^-/-^ mice demonstrated significantly lower cell infiltration in the ischemic hemisphere as compared to WT mice (*P* ≤0.05). However, there was no difference in leukocyte numbers between the ischemic brains of the PD-L1^-/-^ and PD-L2^-/-^ mice (Figure 2A).

Since PD-L1^-/-^ mice demonstrated significantly less severe clinical and histological signs of MCAO after 96 hours of reperfusion, we evaluated the immune cell trafficking into the CNS of these mice compared to WT and PD-L2^-/-^ mice. Mononuclear cells, isolated from ischemic and non-ischemic hemispheres of brains of WT, PD-L1^-/-^, and PD-L2^-/-^ mice after 96 hours of reperfusion, were stained for several cell surface markers. Upon characterizing infiltrating mononuclear cells in the brains, it was revealed that ischemic brain hemispheres of WT (*P* ≤0.001) and PD-L2^-/-^ mice (*P* ≤0.05) had significantly higher numbers of microglia and/or infiltrating monocytes/macrophages as compared to their respective non-ischemic halves, while there was no increase in these cells in the ischemic brains of the PD-L1^-/-^ mice. The ischemic hemispheres of the PD-L1^-/-^ mice, in fact, had significantly lower numbers of CD11b^+^CD45^high^ population as compared to the ischemic halves of WT (*P* ≤0.001) and PD-L2^-/-^ (*P* ≤0.05) mice (Figure 2B). Similarly, although there was a significant increase in the numbers of CD4^+^ T cells in the ischemic hemispheres of WT (*P* ≤0.001) and PDL2^-/-^ mice (*P* ≤0.01) as compared to their non-ischemic halves, there were no differences in the PD-L1^-/-^ mice post-MCAO. The ischemic hemispheres of the PD-L1^-/-^ had significantly lower CD4^+^ T cells (*P* ≤0.05) as compared to WT mice (Figure 2C). Both of these populations have been known to accumulate by day 3 postischemic brain injury leading to subsequent production of proinflammatory cytokines and upregulation of cell adhesion molecules [4]. However, when the total CD8^+^ T cell population was analyzed in the brain, we observed a significant increase of CD8^+^ T cell numbers not only in the right hemispheres of the PD-L1^-/-^ mice (*P* ≤0.05) as compared to both WT and PD-L2^-/-^ mice, but also significantly more (*P* ≤0.05) in the left hemisphere of the PD-L1^-/-^ mice as compared to the PD-L2^-/-^ mice post-MCAO (Figure 2D). Thus, with lower infarct volumes, infiltration of inflammatory cells into the CNS was also abrogated in the absence of PD-L1, whereas they were retained in the absence of PD-L2.

Furthermore, TNF-α production by CD11b^+^CD45^high^ subpopulation (Figure 2E) and CD3^+^ T cells (Figure 2F) was determined. The TNF-α production was significantly higher in the T cells of ischemic hemisphere of the brains of WT mice compared to its contralateral hemisphere (*P* <0.01) and also as compared to the ischemic hemisphere of PD-L1^-/-^ mice (*P* <0.05). The ischemic hemispheres of the PD-L2^-/-^ mice also had a significantly higher percentage of TNF-α-producing CD3^+^ T cells as compared to its contralateral side (*P* ≤0.05) and as compared to the ischemic hemisphere of PD-L1^-/-^ mice (*P* ≤0.01) (Figure 2E), further confirming the reduction in inflammatory milieu in the absence of PD-L1.

### Absence of PD-L1 rescues MCAO-induced splenic atrophy and inhibits activation states of splenic T cells and monocytes

In lieu of the unexpected decreased infarct volumes in the PD-L1^-/-^ and PD-L2^-/-^ mice, the peripheral immune status was assessed in sham-treated WT, PD-L1^-/-^, and PD-L2^-/-^ mice to ascertain if there were any existing differences in immune parameters before MCAO that could affect infarct volume following MCAO. Sham MCAO surgeries were performed in each mouse strain by placing the intraluminal filament but not advancing it to achieve MCAO. However, as illustrated in Tables 2A and 3A, no significant differences either in absolute numbers of splenocytes or any of the immune parameters analyzed were observed. Since our laboratory has previously established a stroke-induced splenic atrophy phenomenon [2], splenic cell numbers in each of the WT, PD-L1^-^/^-^, and PD-L2^-^/^-^ mice were evaluated at 96 hours post-MCAO. As expected and previously demonstrated [2], MCAO resulted in large reductions in spleen cell numbers in WT mice. The reduction in spleen counts was from approximately 80 million spleen cells/sham WT mouse (data in Table 2A) to approximately 9 million spleens cells/WT mouse after 60 minutes of MCAO and 96 hours of reperfusion, accounting for approximately 89% reduction in the total splenocytes (Table 2B). Interestingly, the spleens of PD-L1^-/-^ mice did not sustain such marked atrophy in the splenic numbers (accounting for approximately 73% reduction as compared to its sham-treated group) (Table 2B). These viable cell counts in MCAO-induced PD-L1^-/-^ mice were significantly higher than those of the MCAO-induced WT mice (*P* <0.05). While the spleens of PD-L2^-/-^ underwent an approximately 80% reduction from their sham state, this change was not significant when compared to the viable splenic cell numbers from either MCAO-induced WT or PD-L1^-/-^ mice (Table 2B).

**Table 2:** **Spleen cell yield from sham and MCAO-subjected WT, PD-L1**^**-/-**^**, and PD-L2**^**-/- **^**mice**

**A**			**B**		
**Cell yield (**× **10**^**6**^**)**			**Cell yield (**× **10**^**6**^**)**		
**WT**	**PD-L1**^**-/-**^	**PD-L2**^**-/-**^	**WT**	**PD-L1**^**-/-**^	**PD-L2**^**-/-**^
**Sham (n)**	**Sham (n)**	**Sham (n)**	**MCAO (n)**	**MCAO (n)**	**MCAO (n)**
79.9 ± 4.5 (9)	85.6 ± 3.1 (13)	94.3 ± 3.6 (10)	8.9 ± 7.3 (12)^a,b^	23.1 ± 3.5 (19)^a^	19.5 ± 1.9 (14)^a^

Data are presented as mean ± SEM. ^a^Value of *P* ≤0.05 with respect to the strain’s sham group; ^b^value of *P* ≤0.05 versus MCAO-induced PD-L1^-/-^ mice. ^-^/^-^, knockout; MCAO, middle cerebral artery occlusion; PD-L1, programmed death-1 ligand 1; PD-L2, programmed death-1 ligand 2; WT, wild-type.

**Table 3:** **Percentage of different cell types in spleens of WT, PD-L1**^**-/-**^**, and PD-L2**^**-/- **^**male mice 96 hours after MCAO**

**A**				**B**			
**Cell types (%)**				**Cell types (%)**			
**Cell type**	**WT**	**PD-L1**^**-/-**^	**PD-L2**^**-/-**^	**Cell type**	**WT**	**PD-L1**^**-/-**^	**PD-L2**^**-/-**^
	**Sham (n)**	**Sham (n)**	**Sham (n)**		**MCAO (n)**	**MCAO (n)**	**MCAO (n)**
CD4	20.7 ± 3.9 (7)	21.5 ± 2.5 (9)	17.7 ± 3.1 (8)	CD4	25.4 ± 4.2 (8)^b^	32.8 ± 6.2 (9)^a^	25.0 ± 6.5 (7)^a,b^
CD8	8.9 ± 3.1 (7)	11.4 ± 2.3 (9)	9.4 ± 2.3 (8)	CD8	13.9 ± 6.0 (8)^b^	25.0 ± 8.3 (9)^a^	21.3 ± 5.4 (7)^a^
CD19	64.8 ± 7.3 (7)	60.0 ± 4.0 (9)	65.5 ± 2.9 (8)	CD19	53.7 ± 9.0 (8)^b^	40.3 ± 7.7 (11)^a^	43.2 ± 12.5 (7)^a^
CD11b	2.3 ± 0.9 (7)	4.4 ± 1.8 (9)	3.3 ± 0.6 (8)	CD11b	2.0 ± 0.7 (9)	3.2 ± 2.1 (9)	1.9 ± 1.5 (7)

Data are presented as mean ± SEM. ^a^Value of *P* ≤0.05 with respect to the strain’s sham group; ^b^value of *P* ≤0.05 versus MCAO-induced PD-L1^-/-^ mice. ^-^/^-^, knockout; MCAO, middle cerebral artery occlusion; PD-L1, programmed death-1 ligand 1; PD-L2, programmed death-1 ligand 2; WT, wild-type.

Given the partial restoration of splenic cell numbers in the MCAO-induced PD-L1^-/-^ and PD-L2^-/-^ mice, we further evaluated specific surviving splenic cell types. Percentages of T cells (CD4^+^ T cells and CD8^+^ T cells), B cells, and monocytes were enumerated. As previously determined [2], the WT mice post-MCAO demonstrated an increase in the percentages of CD4^+^ T cells and CD8^+^ T cells, with a reduction in the B cell percentages (Table 3B). Similarly, there was a significant increase in the percentage of T cells (CD4^+^ and CD8^+^) in both the MCAO-induced PD-L1^-/-^, and in the MCAO-induced PD-L2^-/-^ as compared to their sham-treated counterparts (Table 3A,B). Moreover the percentages of T cells (CD4^+^ and CD8^+^) in the spleens of MCAO-induced PD-L1^-/-^ mice were significantly higher than those in the MCAO-subjected WT, with no significant difference between the WT and PD-L2^-/-^ cell composition (Table 3B). The PD-L knockout strains had a substantial loss of B cell percentages from their spleens not only when compared to their sham counterparts but also with the MCAO-induced WT mice. No differences in the percentage of monocytes were determined between strains or within treatment conditions (Table 3A,B). However, in spite of these changes in percentages, there remained higher absolute numbers of T cells, B cells, and monocytes in the PD-L1^-/-^ and PD-L2^-/-^ mice with MCAO compared to WT mice (data calculated from Tables 2 and 3), indicating relative preservation of all cell types in PD-L-deficient mice.

At this point, it was unclear whether increased accumulation of CD4^+^ and CD8^+^ T cells in PD-L1^-/-^ mice was due to decreased apoptosis or increased proliferation of T cells. To further ascertain, splenocytes were evaluated for the expression of PI, with PI^+^ cells indicating dying splenocytes. As indicated in Table 4, the PD-L1^-/-^ mice had a significant reduction in the percentage of PI^+^ cells at 96 hours after reperfusion as compared to the MCAO-induced WT mice. The MCAO-subjected PD-L1^-/-^ mice also demonstrated more T cell proliferative responses as compared to the WT mice, whereas the T cell proliferative responses of the PD-L2^-/-^ mice were comparable to those of the MCAO-induced WT mice (data not shown). Thus, these results clearly demonstrate that the presence of PD-L1 contributes more to MCAO-induced cell death and less proliferative responses than PD-L2 after 96 hours of ischemic stroke in mice.

**Table 4:** **Percentage of dead (propidium iodide (PI**^**+**^**)) cells in spleens of WT, PD-L1**^**-/-**^**, and PD-L2**^**-/- **^**male mice 96 hours after MCAO**

**Dead cells (PI**^**+ **^**%)**		
**WT**	**PD-L1**^**-/-**^	**PD-L2**^**-/-**^
MCAO (n)	MCAO (n)	MCAO (n)
24.3 ± 6.4 (12)^a^	18.3 ± 2.4 (19)	20.2 ± 3.9 (14)

Data are presented as mean ± SEM. ^a^Value of *P* ≤0.05 versus MCAO-induced PD-L1^-/-^ mice. ^-^/^-^, knockout; MCAO, middle cerebral artery occlusion; PD-L1, programmed death-1 ligand 1; PD-L2, programmed death-1 ligand 2; PI, propidium iodide; WT, wild-type.

**Figure 3:**
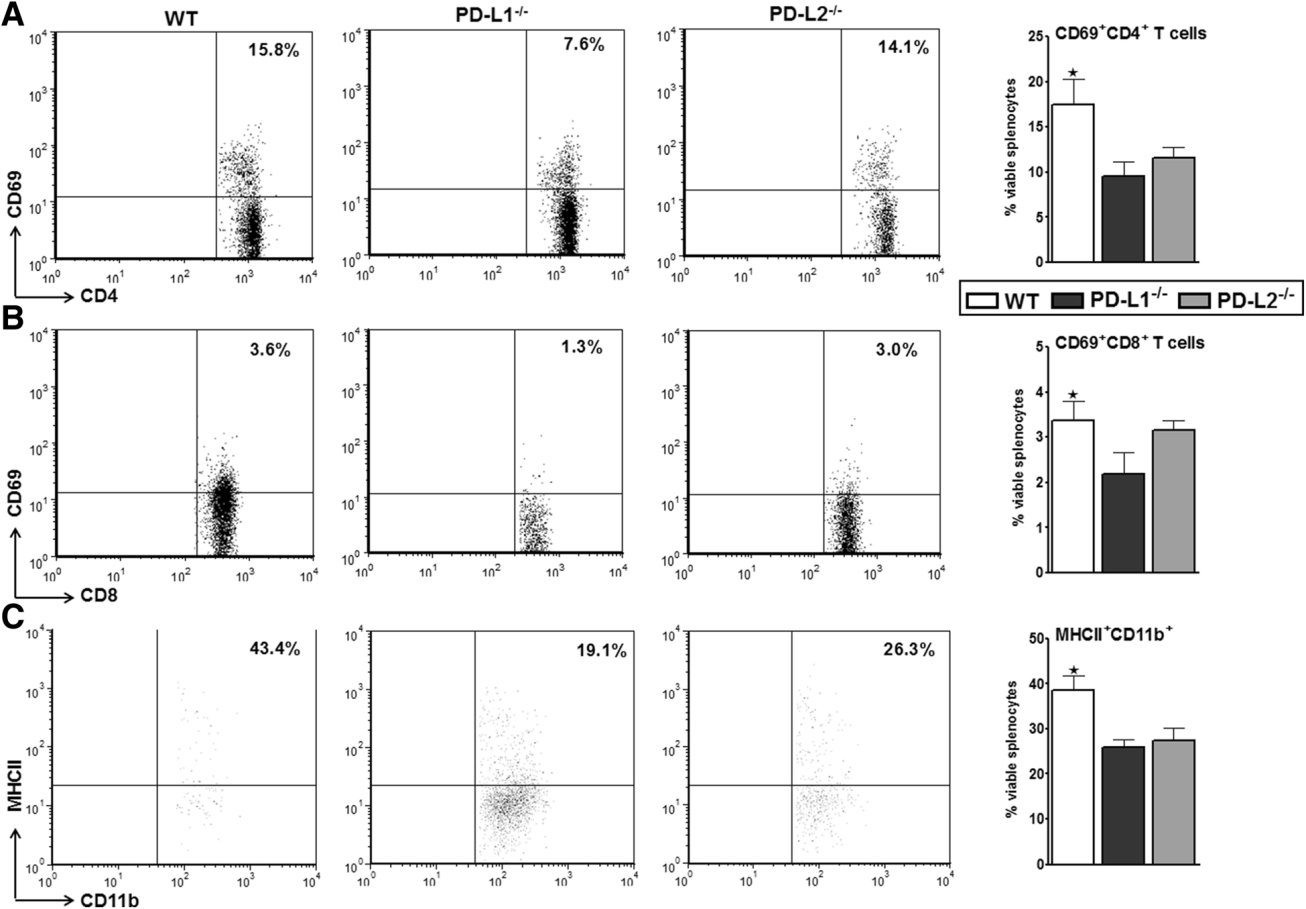
**Absence of PD-L1 inhibited activation states of splenic T cells and monocytes post-MCAO.** Ninety six hours after MCAO, mononuclear cells were isolated from spleens of WT, PD-L1^-/-^, and PD-L2^-/-^ mice and analyzed. **(A)** Expression of activation marker CD69 on gated CD4^+^ T cells. **(B)** Expression of activation marker CD69 on gated CD8^+^ T cells. **(C)** Expression of MHC class II by CD11b^+^ monocytes. Values represent mean numbers (± SEM) of indicated cell subsets, gated on live leukocytes (by PI exclusion) from seven to eight mice in each group, from at least three separate experiments. Statistical analysis was performed with ANOVA followed by Tukey’s multiple comparison post-hoc test. Significant differences between sample means are indicated. *P ≤0.05 compared to the PD-L1^-/-^ mice post-MCAO. ^-^/^-^, knockout; ANOVA, analysis of variance; MCAO, middle cerebral artery occlusion; MHC, major histocompatibility complex; PD-L1, programmed death-1 ligand 1; PD-L2, programmed death-1 ligand 2; PI, propidium iodide; SEM, standard error of the mean; WT, wild-type.

To further evaluate the possible differences due to the loss of the PD-Ls that could have led to attenuated stroke outcome, activation states of T cells and monocytes were quantified in mice subjected to 60 minutes of MCAO and 96 hours of reperfusion. Thus, to correlate the reduced infarct volumes and reduced splenic atrophy to the activation states of T cells in the spleens which might be influencing the splenic milieu, we evaluated the percentage expression of CD69 by CD4^+^ T cells and CD8^+^ T cells, and of major histocompatibility complex (MHC) class II by CD11b^+^ monocytes in the spleens of MCAO-induced WT, PD-L1^-/-^, and PD-L2^-/-^ mice. CD69 is an ‘activation marker’ that is rapidly induced on mature T cells after stimulation through the TCR. As shown in Figures 3A,B, splenocytes from PD-L1^-/-^ mice after MCAO had significantly decreased percentages of CD69^+^CD4^+^ (*P* ≤0.05) and CD69^+^CD8^+^ (*P* ≤0.05) T cells as compared to the MCAO-induced WT mice. The activation states of T cells (CD4^+^ and CD8^+^) in the PD-L2^-/-^ mice were slightly reduced as compared to the WT mice after MCAO, but not significantly. Similarly, the expression of MHC class II on monocytes was significantly reduced (*P* ≤0.05) in the PD-L1^-/-^ mice (Figure 3C) as compared to those in MCAO-induced WT mice. These results indicate that PD-L1 and to a lesser extent PD-L2 facilitates the increased activation of T cells and monocytes at 96 hours post-MCAO.

### Absence of PD-L1 leads to increased expression of PD-1 on T cells with no difference in Foxp3^+^ T cells in the spleens

**Figure 4:**
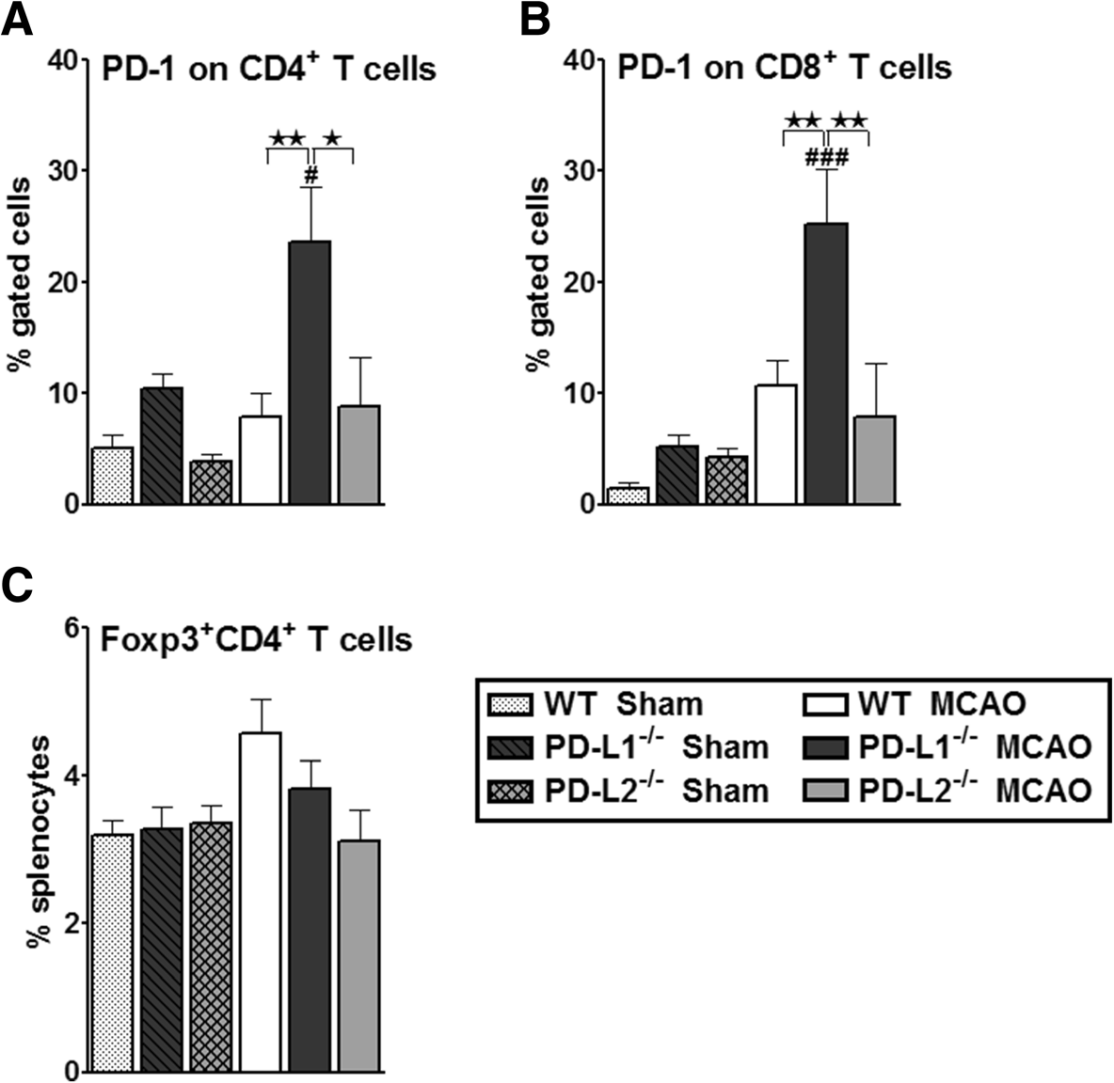
**Absence of PD-L1 led to increased expression of PD-1 on T cells with no difference in Foxp3**^**+ **^**T cells in the spleens.** Splenocytes from WT, PD-L1^-/-^, and PD-L2^-/-^ mice were harvested at 96 hours post-MCAO or sham-treatment and assessed for expression. **(A)** PD-1 expression on gated CD4^+^ T cells. **(B)** PD-1 expression on gated CD8^+^ T cells. **(C)** Expression of Foxp3^+^CD4^+^ T cells. Values represent mean numbers (± SEM) of indicated cell subsets from four to five mice of each group, from two separate experiments. Statistical analysis was performed with ANOVA followed by Tukey’s multiple comparison post-hoc test. Significant differences between sample means are indicated (#*P* ≤0.05 and ###*P* ≤0.001 as compared to their respective sham;*P ≤0.05 and **P ≤0.01 compared to the PD-L1^-/-^ mice post-MCAO). ^-^/^-^, knockout; ANOVA, analysis of variance; MCAO, middle cerebral artery occlusion; PD-1, programmed death-1; PD-L1, programmed death-1 ligand 1; PD-L2, programmed death-1 ligand 2; SEM, standard error of the mean; WT, wild-type.

Since our previous work [22] implicated PD-1 signaling in limiting CNS inflammation in MCAO, we next sought to assess the expression of this co-inhibitory receptor on the splenic T cells from each of the three mouse strains post-MCAO. Interestingly, there was a significant increase in percentage of PD-1 expression on both splenic CD4^+^ and CD8^+^ T cells of the PD-L1^-/-^ mice at 96 hours post-MCAO (Figure 4A,B). This increase was significant as compared to both the WT mice (*P* ≤0.01) for CD4^+^ and CD8^+^ T cells, and also to the PD-L2^-/-^ mice (*P* ≤0.05) for CD4^+^ T cells and (*P* ≤0.01) for CD8^+^ T cells. However, there were no differences in the expression of PD-1 among strains of the sham-treated mice. Our earlier studies demonstrated that MCAO induced a threefold increase in the percentage of Foxp3^+^CD4^+^ Tregs in the spleens as compared to sham or naive mice [2]. Also, a role for the PD-1/PD-L pathway in generation of Tregs has been supported by a number of studies [1,8]. Hence, the levels of Foxp3^+^CD4^+^ T cells were assessed in each of the three mouse strains, post-MCAO. The PD-L-deficient mice demonstrated a trend towards lower expression of Foxp3^+^CD4^+^ T cells although these levels were not significant as compared to those in the WT mice at 96 hours post-MCAO (Figure 4C). These results still indicate that PD-1 as demonstrated earlier [7] might play a crucial role through its interaction with another ligand, predominantly PD-L2, in the absence of PD-L1, leading to decreased activation states of the T cells at 96 hours post-MCAO.

### Absence of PD-L1 results in loss of suppressor T cells from spleens

**Figure 5:**
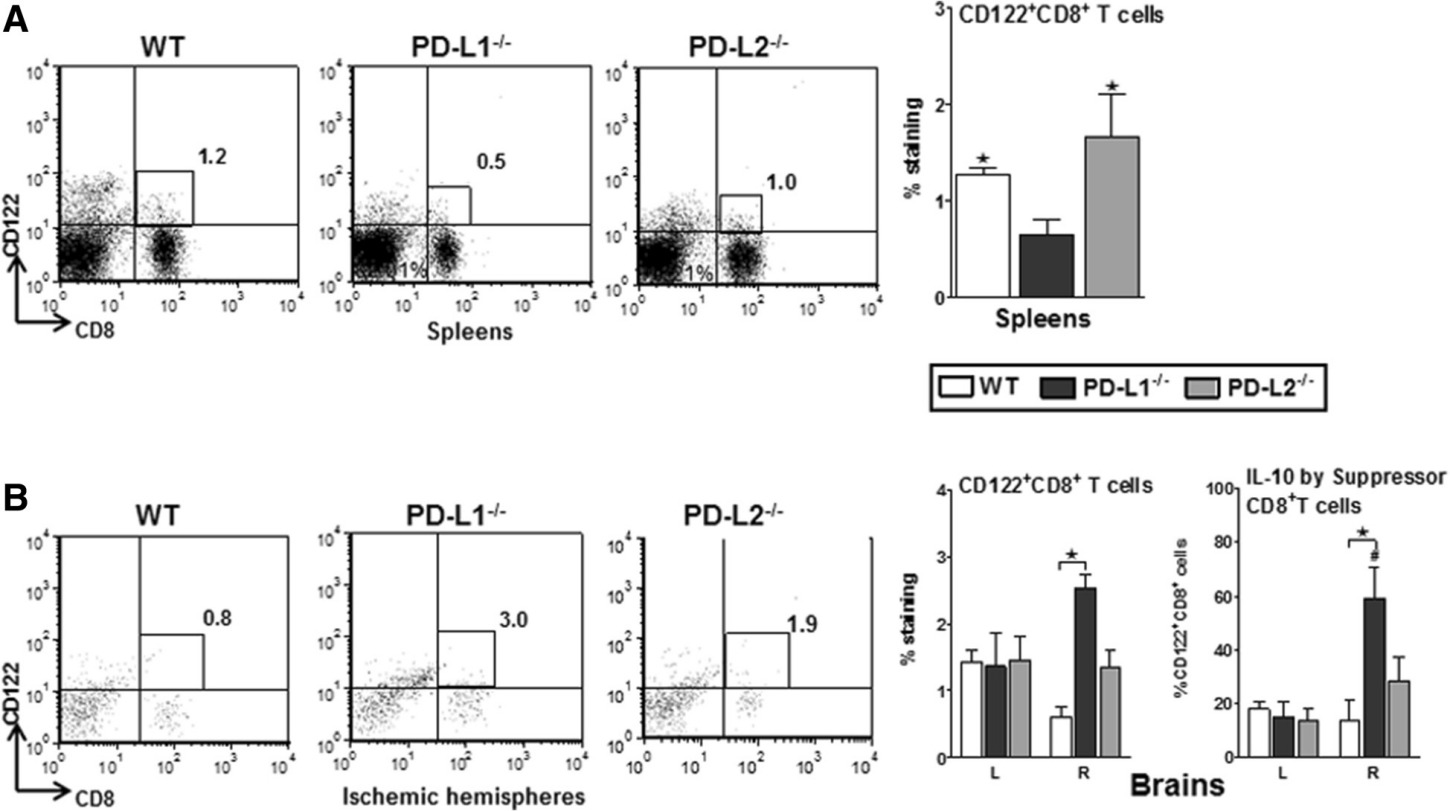
**Absence of PD-L1 resulted in loss of suppressor T cells from spleens.** Splenocytes from WT, PD-L1^-/-^, and PD-L2^-/-^ mice were harvested at 96 hours post-MCAO and assessed. **(A)** Expression of CD8^+^CD122^+^ T cells. Data are representative of two independent experiments with spleens processed from four to five individual mice (mean ± SEM). Statistical analysis was performed with ANOVA followed by Tukey’s multiple comparison post-hoc test. Significant differences between sample means are indicated (*P ≤0.05 compared to the PD-L1^-/-^ mice post-MCAO). Subsequently, mononuclear cells were isolated from brains of WT, PD-L1^-/-^, and PD-L2^-/-^ male mice and analyzed. **(B)** CD8^+^CD122^+^ T cells and IL-10 production by gated CD8^+^CD122^+^ T cells. Data are representative of two independent experiments with spleens processed from four to five individual mice (mean ± SEM). Statistical analysis was performed with ANOVA followed by Tukey’s multiple comparison post-hoc test. Significant differences between sample means are indicated (#*P* ≤0.05 as compared to their respective left hemisphere and *P ≤0.05 as compared to the ischemic right hemisphere of PD-L1^-/-^ mice post-MCAO). ^-^/^-^, knockout; ANOVA, analysis of variance; MCAO, middle cerebral artery occlusion; PD-L1, programmed death-1 ligand 1; PD-L2, programmed death-1 ligand 2; SEM, standard error of the mean; WT, wild-type.

To further evaluate possible regulatory parameters, we assessed the expression of a recently characterized regulatory T cell (Treg) sub-population known as CD8^+^CD122^+^ suppressor T cells [29]. CD8^+^CD122^+^ Tregs are naturally occurring Tregs that effectively suppress the proliferation and IFN-γ production of both CD8^+^ and CD4^+^ target cells by virtue of their IL-10 production [30]. A significant decrease in the percentage of CD8^+^CD122^+^ Tregs in the PD-L1^-/-^ mice both as compared to the WT (*P* ≤0.05) and the PD-L2^-/-^ (*P* ≤0.05) mice is shown in Figure 5A. Upon trying to rationalize this decrease in CD8^+^CD122^+^ T cells from the spleens, low infarcts in the PD-L1^-/-^ mice and increased numbers of total CD8^+^ T cells in the ischemic hemispheres of PD-L1^-/-^ mice, we speculated a plausible exit of these suppressors from the spleens and their entry into the ischemic hemisphere of the brains. Upon further characterizing the CD8^+^ T cells in the brains for the presence of the suppressor CD8^+^ T cell sub-population, there was indeed a significant increase in the percentage of CD8^+^CD122^+^ T cells in the ischemic hemispheres of the PD-L1^-/-^ mice as compared to those found in the ischemic hemispheres of the WT mice (*P* ≤0.05) after 96 hours of reperfusion (Figure 5B). Not only were the percentages of the suppressor CD8^+^ T cells higher, but they also expressed higher IL-10 levels in the ischemic half of the brains of PD-L1^-/-^ mice. These data strongly support the contention that in the absence of PD-L1, the suppressor T cells are able to exit the periphery and enter the CNS, thereby contributing to a plausible immunomodulation following stroke and thereby preventing further damage from pro-inflammatory factors after the 96-hour timeframe.

### Absence of PD-L1 leads to decreased expression of CD80 on APCs in spleens

**Figure 6:**
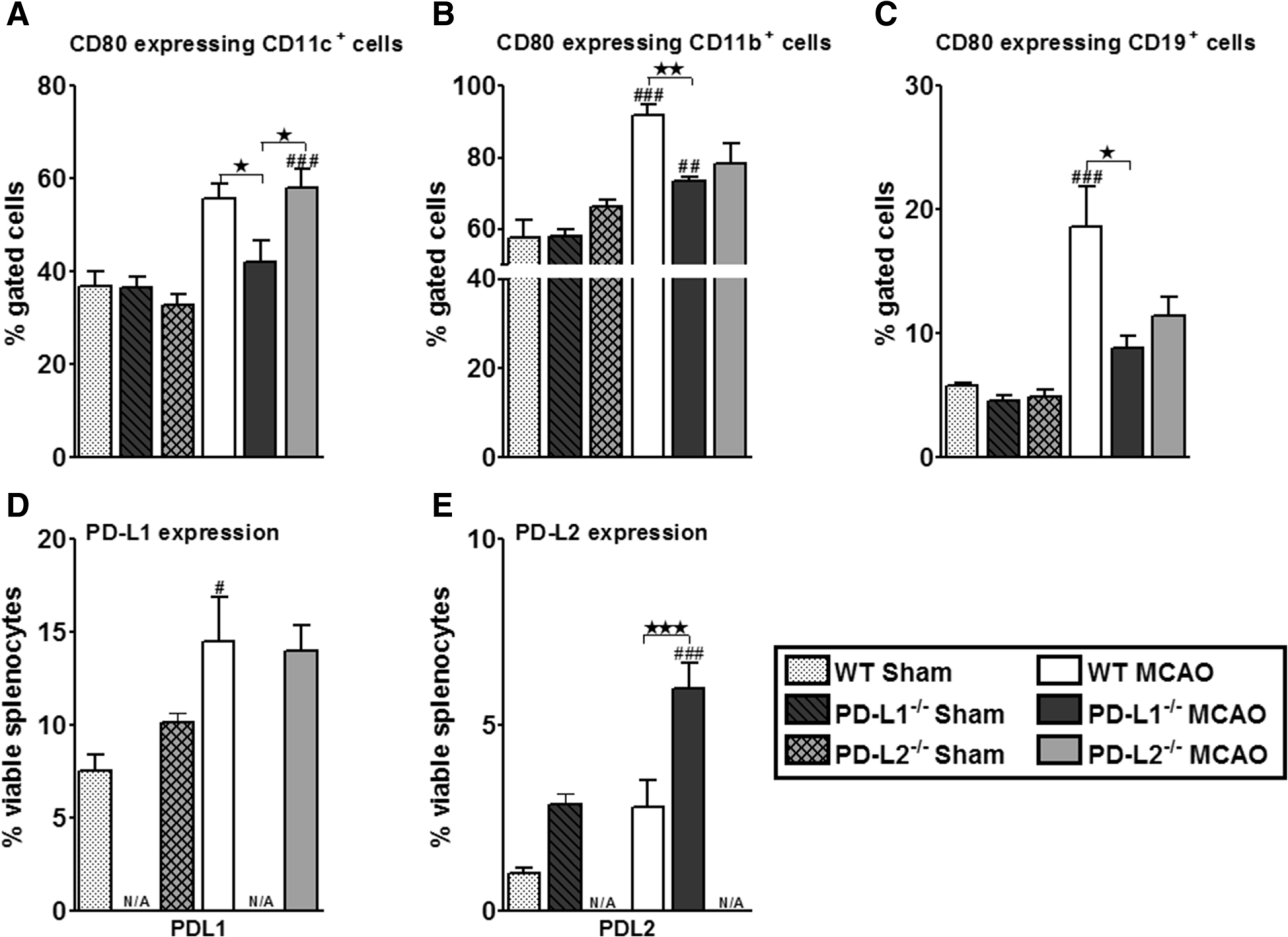
**Absence of PD-L1 led to decreased expression of CD80 on APCs in spleens post-MCAO.** Splenocytes from WT, PD-L1^-/-^, and PD-L2^-/-^ mice were harvested at 96 hours post-MCAO or sham-treatment and assessed for expression. CD80 expression on gated **(A)** CD11c^+^, **(B)** CD11b^+^, and **(C)** CD19^+^ cells. **(D)** Expression of total PD-L1 and **(E)** PD-L2 on leukocytes. Values represent mean numbers (± SEM) of indicated cell subsets, gated on live leukocytes (by PI exclusion) from four to five mice of each group, from two separate experiments. Statistical analysis was performed with ANOVA followed by Tukey’s multiple comparison post-hoc test. Significant differences between sample means are indicated (#*P* ≤0.05; ##*P* ≤0.01; ###*P* ≤0.001 as compared to their respective sham; *P ≤0.05 and **P ≤0.01 compared to the PD-L1^-/-^ mice post-MCAO). ^-^/^-^, knockout; ANOVA, analysis of variance; APC, antigen-presenting cell; MCAO, middle cerebral artery occlusion; PD-L1, programmed death-1 ligand 1; PD-L2, programmed death-1 ligand 2; PI, propidium iodide; SEM, standard error of the mean; WT, wild-type.

Recent studies demonstrated that PD-L1 binds CD80 (B7-1) besides PD-1, and PD-L1/CD80 interaction also delivers inhibitory signals in T cells [31,32]. Hence, in our attempt to address this mechanistic possibility, we analyzed CD80 expression on the APCs in the periphery. As demonstrated in Figure 6A, there was a significant reduction in CD80 expression on the CD11c^+^ DCs in the PD-L1^-/-^ mice, both as compared to the WT (*P* ≤0.05) and PD-L2^-/-^ (*P* ≤0.05) mice, and by the CD11b^+^ monocytes (*P* ≤0.01) (Figure 6B) and CD19^+^ B cells (*P* ≤0.05) (Figure 6C) as compared to the WT mice post-MCAO. There was no difference in the CD80 expression on CD11c^+^, CD11b^+^, and CD19^+^ cells in sham MCAO conditions. The CD80 expressing CD11b^+^ and CD19^+^ cells increased significantly (*P* ≤0.001) in the WT mice post-MCAO as compared to the shams. In the PD-L1^-/-^ and PD-L2^-/-^ mice, CD80 expression on CD11b^+^ cells (*P* ≤0.01) and CD80 expression on CD11c^+^ cells increased significantly (*P* ≤0.001), respectively, post-MCAO, as compared to their sham-treated counterparts. However, the PD-L1^-/-^ mice, as demonstrated in Figure 4A, also increased PD-1 expression on the T cells. In light of this finding and the reduced expression of CD80 on the APCs, we hypothesized that in the absence of PD-L1, PD-L2 being the only available co-inhibitory ligand for PD-1, the PD-1/PD-L2 interaction might also lead to co-inhibition. Hence, we determined the total PD-L1 and PD-L2 expression on leukocytes in sham and MCAO-subjected mice. As demonstrated in Figure 6D, although expression levels of PD-L1 in the WT mice increased post-MCAO as compared to its sham (*P* ≤0.05), there was no difference in the PD-L1 expression between the WT and PD-L2^-/-^ mice after MCAO, with no difference between the groups in sham conditions. However, there was indeed an increase in the total PD-L2 expression in the PD-L1^-/-^ mice as compared to the WT mice (*P* ≤0.001) post-MCAO (Figure 6E), making a plausible case for a PD-1/PD-L2 co-inhibitory interaction.

## Discussion

Ischemic stroke induces neurological deficits in almost one-third of patients, leading to increased mortality and long-term functional disability [33,34]. Although underlying mechanisms have not been completely unraveled, that ischemia evokes inflammatory responses has been characterized. This process depends in part on T cell activation, in which the B7 family of co-stimulatory molecules plays a pivotal role. T cells are localized in close vicinity to blood vessels in the infarct boundary as early as 24 hours after experimental focal cerebral ischemia in rodents [8,35]. This early (24-hour) appearance of T cell infiltration into the brain after MCAO [8,35] may indicate that recruitment of activated cells is antigen-nonspecific, perhaps generated by sympathetic signaling from the brain to the periphery. Alternatively, leakage of brain antigens across a compromised blood brain barrier could initiate a peripheral immune response. Activated T cells have the capacity to infiltrate the brain, and could contribute to expansion of the ischemic penumbra, an area that already contains infiltrating neutrophils after 24 hours. In our previous studies in severe combined immunodeficiency (SCID) mice examining the hypothesis that activated peripheral T cells and B cells would home to the injured brain and alter the evolving infarct, we found that both cortical and total hemispheric infarct volumes were strikingly reduced at 22 hours after MCAO [36], thus indicating a damaging effect of T cells and B cells on early evolving ischemic brain injury. However, subsequent studies have convincingly demonstrated the regulatory role played by the B cells in our model of ischemic stroke [3,9]. Thus, this shifted the focus on further discerning the role of pathogenic T cells. The outcome of effector T cells is decided by the interaction of co-stimulatory molecules on T cells and APCs. Studies in the transient MCAO model [37] point to the non-essentiality of the accessory TCR signaling via the PD-1/PD-L1 or CD28-B7 pathway in early stroke progression. However, since the end-point of these studies was as early as 22 hours post-stroke, it was necessary to investigate the role of the molecules that comprise the co-stimulatory pathway in T cell activation beyond the 22-hour post-MCAO window. Moreover, it is known that inflammation plays an important role in the pathogenesis of ischemic stroke, with stroke patients with systemic inflammation exhibiting clinically poorer outcomes [38,39]. Hence, in our earlier studies, we determined the role of the receptor, PD-1, known to be a part of a co-inhibitory pathway in T cell stimulation using PD-1^-/-^ mice which were subjected to 60 minutes of MCAO followed by 96 hours of reperfusion. These studies demonstrated a critical role for PD-1 in limiting functional and histological damage after MCAO [7]. However the role of the two PD-Ls was not known. Therefore, to complete the picture, it was necessary to investigate the nature of interactions between PD-1 and both its ligands for understanding the susceptibility, pathogenic mechanisms, and protection afforded after ischemic stroke. Hence, the same time point of reperfusion, as that followed for our earlier studies in PD-1^-/-^ mice, was selected as a starting point of discerning the effects of interaction between PD-1 and its ligands in ischemic stroke.

In our current study, we hypothesized a critical role for the PD-Ls in exacerbating ischemic stroke. However, as clearly demonstrated in Figures 1 and 2, the loss of PD-L1 and to a lesser extent PD-L2 resulted in better stroke outcomes and decreased infiltration of immune cells in the ischemic hemisphere of PD-L1^-/-^ mice. The PD-1/PD-L pathway is recognized to control peripheral T cell tolerance in several ways. This pathway can limit the initial phase of activation and expansion of self-reactive T cells, and restrict self-reactive T cell effector function. The PD-1/PD-L interactions also play a role in inhibiting expansion of naive self-reactive T cells and/or their differentiation into effector T cells [11]. Thus, PD-L1 and PD-L2 can signal bidirectionally by engaging PD-1 on T cells and by delivering signals into PD-L-expressing cells. Although PD-L1 has been consistently shown to be inhibitory in secondary phases of immune reactions [40,41], its role in primary immune reactions is less clear. Initial reports have attributed both stimulatory and inhibitory properties under conditions of primary immune reactions [22]. However, a number of recent studies suggest a ‘proautoimmune’ role for PD-L1 rather than a suppressive function. For example, transgenic over-expression of PD-L1 on pancreatic beta cells enhanced autoimmunity instead of suppressing it [42]. As a result of PD-L1 over-expression in beta cells, CD8^+^ T cell proliferation was enhanced and immunological tolerance was broken, as mice developed spontaneous diabetes [42]. In yet another study [43], an unexpected beneficial effect from PD-L1^-/-^ DCs was demonstrated where intracerebral microinjections resulted in amelioration of subsequent experimental autoimmune encephalomyelitis (EAE) [43]. Furthermore, this treatment was accompanied by amplified neuroantigen-specific CD8^+^ Treg recruitment into the CNS, suggesting that the lack of DC-derived PD-L1 allows for the development (or recruitment) of regulatory CD8^+^ T cells (CD122^+^) in the CNS. Thus, by ‘inhibiting the inhibitors’, DC/APC expression of PD-L1 at the site of inflammation leads to exacerbated autoimmunity [44]. Our finding in this current study match this aforementioned scenario, since as demonstrated in Figure 5A,B, there was a loss of the suppressor CD8^+^ subpopulation from the periphery post-MCAO in the PD-L1^-/-^ mice, only to find its accumulation in the ischemic half of the brains.

A number of lines of evidence have suggested a receptor for PD-L1 (B7-H1) or PD-L2 (B7-DC), aside from PD-1. CD80 (B7-1) has recently been identified as a binding partner for PD-L1 [11]. Surface plasmon resonance studies demonstrate specific and unique interaction between PD-L1 and CD80, with an affinity (approximately 1.7 μM) intermediate between the affinities of CD80 for CD28 (4 μM) and CTLA-4 (0.2 μM), and PD-L1 for PD-1 (0.5 μM). CD86 (B7-2) does not bind to PD-L1 or PD-L2, and PD-L2 does not bind to CD80. CD80/PD-L1 interactions can induce an inhibitory signal into T cells. Ligation of PD-L1 on CD4 T cells by CD80, or ligation of CD80 on CD4 T cells by PD-L1, delivers a functionally significant inhibitory signal. CD80 acts specifically through PD-L1 on the T cell in the absence of CD28 and CTLA-4. Thus, PD-L1 can exert an inhibitory effect on T cells either through CD80 or PD-1. Because both PD-L1 and CD80 are expressed on T cells, B cells, DCs, and macrophages, there is the potential for bidirectional interactions between CD80 and PD-L1 on these cell types. In addition, PD-L1 on nonhematopoietic cells may interact with CD80 as well as PD-1 on T cells to regulate cells. In this scenario, PD-L1 binds CD80 to compete off CD28 binding due to its higher affinity interaction. Not mutually exclusive, PD-L1 might also be required as a survival signal for brain-derived CD8^+^ Tregs. Thus, as demonstrated in Figure 6A,B,C in our present study, an increased expression of CD80 on APCs in the WT and PD-L2^-/-^ mice might suggest an overriding CD80/CD28 interaction leading to T cell activation. Conversely, low CD80 expression in PD-L1^-/-^ mice and the increase in the total PD-L2 expression in the PD-L1^-/-^ mice as compared to the WT mice makes a plausible case for a PD-1/PD-L2 co-inhibitory interaction in the absence of PD-L1.

However, one also needs to keep in mind that the broad distribution of PD-L1 expression on hematopoietic and parenchymal cells suggests one or both cell types may be central to limiting autoimmune disease during multiple phases of the immune responses. Due to its broad expression pattern in lymphoid and non-lymphoid organs, the PD-L1/PD-1 pathway has been suggested to play a crucial role for the maintenance of immune tolerance [45,46]. In fact, the expression of PD-L1 on vascular endothelial cells has led to the hypothesis that PD-L1 on endothelial cells may regulate the activation of T cells that contact the vessel wall, the extravasation of T cells into tissue, and/or limit detrimental consequences of immunopathology [12]. Therefore, further studies pertaining to the role of PD-L1 on the endothelial cells become necessary in determining the exact role of PD-L1 in ischemic stroke.

The present study has some limitations with regards to outcome evaluations. First, we used a neurological deficit scoring system to evaluate each animal. While this scoring system is sensitive enough to confirm ischemia and consequent ischemic injury in each animal, it may not be sensitive enough for evaluating subtle changes in functional outcomes due to experimental manipulations or treatments. Future follow-up studies will use more sophisticated and sensitive behavioral measures to evaluate functional outcomes at different time points following ischemic insult in PD-L1^-/-^, PD-L2^-/-^, and WT mice. Second, individuals performing infarct volume and immunological assessments were not blinded to genotype. This potentially could have led to subjective bias in assessing results. However, this seems unlikely given that our observed results suggested a pathogenic rather than the expected regulatory role for both PD-Ls. Lastly, differences in striatal infarct volumes between PD-L1^-/-^ and PD-L2^-/-^ mice could be due in part to altered glial proliferative responses as suggested by earlier studies [47]. Evaluation of glial proliferation and the number of microglia and astrocytes surrounding the infarct will be done in future studies.

In summary, the current study conclusively demonstrates for the first time that PD-L1^-/-^ and PD-L2^-/-^ mice have smaller total infarct volume compared to WT mice. Improved stroke outcome was accompanied by significant reduction in the brain infiltrating cells as well as their proinflammatory states in the PD-L1^-/-^ and to a lesser extent PD-L2^-/-^ mice as compared to the WT mice. Our study also demonstrated a novel connection between the ischemic lesion in the brain and evolving inflammatory changes in distant peripheral immune cell populations. Reduction in infarct volumes promoted reduction in ischemia-related splenic atrophy accompanied by lower activated states of the splenic T cells and monocytes in the absence of the PD-L1, suggesting a pathogenic rather than a regulatory role for both PD-Ls. Suppressor T cells (IL-10-producing CD8^+^CD122^+^ T cells) trafficked to the brain in PD-L1^-/-^ mice and their presence in the right MCAO-inflicted hemisphere of the PD-L1^-/-^ mice implicates them as possible key contributors in controlling further adverse effects of ischemia. There was increased expression of CD80 on splenic APCs in WT and PD-L2^-/-^ mice, suggesting an overriding interaction leading to T cell activation. Conversely, low CD80 expression by the APCs in PD-L1^-/-^ mice suggests suppression possibly via PD-1/PD-L2 interactions. Our novel observations are the first to implicate PD-L1 in enhancing the severity of experimental stroke. These results suggest that agents (for example antibodies) that can target and neutralize PD-L1/2 may have therapeutic potential for treatment of human stroke.

## Abbreviations

2-β-ME: 2-β-mercaptoethanol; ANOVA: analysis of variance; APC: antigen-presenting cell; CNS: central nervous system; CTLA-4: cytotoxic T lymphocyte antigen 4; DC: dendritic cell; EAE: experimental autoimmune encephalomyelitis; DNase I: deoxyribonuclease I; FACS: fluorescence-activated cell sorting; FCS: fetal calf serum; Foxp3: forkhead box P3; IFN: interferon; IL: interleukin; LDF: laser Doppler flowmetry; LPS: lipopolysaccharide; mAb: monoclonal antibodies; MCAO: middle cerebral artery occlusion; MHC: major histocompatibility complex; NIH: National Institutes of Health; NKT: natural killer T; PBS: phosphate buffered saline; PD-1: programmed death-1; PD-L: programmed death-1 ligand; PD-L1: programmed death-1 ligand 1; PD-L2: programmed death-1 ligand 2; PI: propidium iodide; PMA: phorbol 12-myristate 13-acetate; RPMI: Roswell Park Memorial Institute; SCID: severe combined immunodeficiency; SEM: standard error of the mean; TCR: T cell receptor; TNF: tumor necrosis factor; Treg: regulatory T cell; WT: wild-type.

## Competing interests

The authors declare that they have no competing financial interests.

## Authors’ contributions

SB performed and interpreted the immunology experiments, carried out statistical analyses, prepared graphics, and wrote the manuscript. YC performed the MCAO procedures, carried out statistical analyses, prepared the graphics, and wrote the methods and results for infarct volume data. AAV critiqued and edited the manuscript. SJM directed study design and data analysis of the MCAO experiments and edited the manuscript. HO directed the overall study, designed and supervised the immunological studies and data analysis, and edited the manuscript. All authors read and approved the final version of the manuscript.
